# Regression-Based Active Learning for Accessible Acceleration of Ultra-Large Library Docking

**DOI:** 10.1021/acs.jcim.3c01661

**Published:** 2023-12-29

**Authors:** Egor Marin, Margarita Kovaleva, Maria Kadukova, Khalid Mustafin, Polina Khorn, Andrey Rogachev, Alexey Mishin, Albert Guskov, Valentin Borshchevskiy

**Affiliations:** † Research Center for Molecular Mechanisms of Aging and Age-related Diseases, Moscow Institute of Physics and Technology, Dolgoprudny 141701, Russia; § University Grenoble Alpes, Inria, CNRS, Grenoble INP, LJK, 38000 Grenoble, France; ∥ Groningen Biomolecular Sciences and Biotechnology Institute, University of Groningen, Nijenborgh 4, 9747 AG Groningen, The Netherlands; ¶ Joint Institute for Nuclear Research, Dubna 141980, Russian Federation

## Abstract

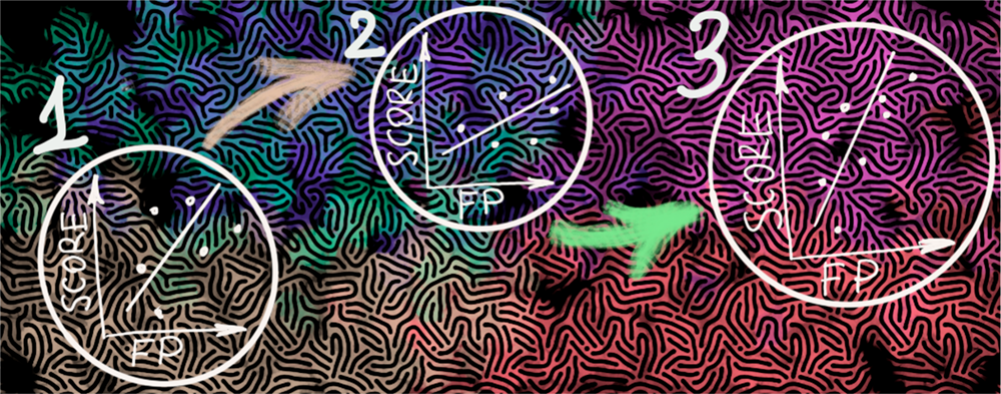

Structure-based drug discovery is a process for both hit finding and optimization that relies on a validated three-dimensional model of a target biomolecule, used to rationalize the structure–function relationship for this particular target. An ultralarge virtual screening approach has emerged recently for rapid discovery of high-affinity hit compounds, but it requires substantial computational resources. This study shows that active learning with simple linear regression models can accelerate virtual screening, retrieving up to 90% of the top-1% of the docking hit list after docking just 10% of the ligands. The results demonstrate that it is unnecessary to use complex models, such as deep learning approaches, to predict the imprecise results of ligand docking with a low sampling depth. Furthermore, we explore active learning meta-parameters and find that constant batch size models with a simple ensembling method provide the best ligand retrieval rate. Finally, our approach is validated on the ultralarge size virtual screening data set, retrieving 70% of the top-0.05% of ligands after screening only 2% of the library. Altogether, this work provides a computationally accessible approach for accelerated virtual screening that can serve as a blueprint for the future design of low-compute agents for exploration of the chemical space via large-scale accelerated docking. With recent breakthroughs in protein structure prediction, this method can significantly increase accessibility for the academic community and aid in the rapid discovery of high-affinity hit compounds for various targets.

## Introduction

Structure-based drug discovery is a process of hit finding and development in target-based drug discovery.^1−3^ It relies on the knowledge of a three-dimensional structure of a verified target, obtained experimentally or modeled. The structure is used for modeling of the drug-target interactions and either for a search for the novel compounds binding the target or for optimization of previously identified binders.

Within this approach, a concept of an ultralarge library docking has recently emerged.^4^ It is used for the rapid discovery of high-affinity hit compounds without iterative optimizations of initial hits. In this approach, an ultralarge (typically, tens of millions of compounds) library is screened against a known target via structure-based docking. Molecules with the best docking score undergo subsequent filtering, and selected compounds (few hundreds) are tested experimentally.^5^ Most studies involve using virtual libraries, such as Enamine REAL.^6^ Their design ensures a high synthesizability rate, thereby providing a sufficiently large number of physically available compounds regardless of the target.

Recently, ultralarge library docking has been shown to provide high-affinity hit compounds for various targets^6−15^ after experimental testing of only few hundreds of compounds. However, such an approach requires substantial computational resources: namely, docking of a single compound requires a few CPU-seconds, resulting in tens of CPU-years for the whole library, which in turn equals to tens of thousands of dollars of computational costs at cloud services such as Google Cloud or Amazon Web Services.^16−18^

Few approaches to reducing computational requirements have been proposed recently. Their main idea is that assignment of precise scores of the whole library is not necessary to retrieve the best scored ones. First, multiple groups have applied active learning for iterative selection of compounds subjected to docking.^19−23^ In the active learning loop, after docking a small batch of ligands, a machine learning (ML) model is trained on the retrieved scores, and the next batch of molecules for docking is chosen based on the predicted docking scores. Binary fingerprints are usually used as features, and prediction of the scores is performed using either deep learning approaches,^15,19,21,23^ gradient boosting,^24^ or simpler models.^20,22^ Furthermore, Graff et al. explored classic recommender system approaches within the active learning framework.^19^

Most of these approaches report a reduction of the computational costs for the docking itself: Gentile et al.^21^ report up to a 100-fold data reduction, Graff et al.^19^ report finding of 95% of the top-50,000 of the library after screening only 2.5% of it, confirmed by Martin^20^ in the later study. Yang et al.^23^ recently reported finding more than 80% of the experimentally confirmed hits with a 14-fold reduction in computational cost. Finally, Luttens et al.^24^ recently reported retrieving >90% of the top-0.004% of the 234 million library after evaluating only 3–5% of ligands.

Finally, most recent approaches^11,13^ rely on the inner structure of ultralarge libraries to substantially reduce the computational requirements in fragment-based manner. Briefly, the building blocks of the ultralarge library are first ranked by their docking score to the target receptor. Then, only compounds that can be synthesized from high-ranked building blocks are subjected to docking. This has led to rapid hit finding for such targets as ROCK1 kinase and human cannabinoid receptors 1 and 2 with docking of less than 1% of the whole library.

In this work, we further explore the active learning approach to accelerate ultralarge structure-based virtual screening. We aim to find a fast base algorithm with good ligand retrieval performance for the active learning loop and then tune the active learning loop parameters, instead of the algorithm itself. To find the base algorithm, we use four different data sets of 1 million molecules. First, we generate data sets of docking scores of 1 million molecules from ZINC20^25^ for two targets, human adenosine receptor A2 (AA2AR) and human cannabinoid receptor 2 (CB2), performing docking with Molsoft ICM. Second, we use existing 1 million subsets of open-access docking score data sets for D4 dopamine receptor (D4) and AmpC β-lactamase (AmpC) from recent studies.^9^

Using these four data sets of docking scores, we benchmark classical ML algorithms in their ability to predict a docking score from molecular fingerprints without any active learning, in a single-iteration mode. In each data set, we label 1% of the highest scored ligands as virtual screening hits (VSHs), and test ML algorithms in their ability to retrieve VSHs from the whole library after training on a subset of ligands. Single-iteration benchmarks show that linear regression can be chosen as a base model for iterative ligand retrieval.

To confirm our choice of the linear regression as a base model, we tested it in the simple active learning regime, finding that a smaller batch size is beneficial for these models. Moreover, we show the inaccuracy of the docking with low sampling depth by using a second docking run as a score predictor and retrieving only 50–70% of the top-1% after screening 10% of the library. After that, we compare multiple active learning regime parameters such as batch size and growth of the training size between iterations. Moreover, we compare different approaches to combine models trained during different iteration steps.

Finally, we compare the best active learning model with models from Graff et al.^19^ using the same AmpC ultra large-scale docking data set.^9^ We show that a simple linear regression ensemble trained on Morgan fingerprints can retrieve 70% of the top-0.05% of the 100 million library after screening only 2% of the ligands. We analyze the chemical diversity of the data set on each iteration using UMAP embeddings^26^ and show that linear regression in active learning regime explores multiple regions of the chemical space.

Altogether, we show that the simplest models such as linear regression, with training and inference time under 1 CPU-minute, perform as base models in the active learning loop on par with more complex regression models such as random forest regression, that take almost three hours for the same task. We conclude that the active learning regime benefits from as small a batch size as 10 000 molecules. For active learning, we find that models with a simple ensembling mechanism confidently outperform other models. These models retrieve 70% of the top-0.05% of the ligands after screening 2% of the whole library, while the best deep learning model reported previously retrieves around 80%, and others retrieve less than 70%.

Overall, we believe that our findings confirm that simple models are as efficient as the previously used deep-learning-based models for predicting the intrinsically inaccurate results of the structure-based molecular docking. We hope that our findings will guide the design of computationally effective accelerated docking pipelines and facilitate rapid drug discovery campaigns using structure-based drug design.

## Materials and Methods

**Figure 1:**
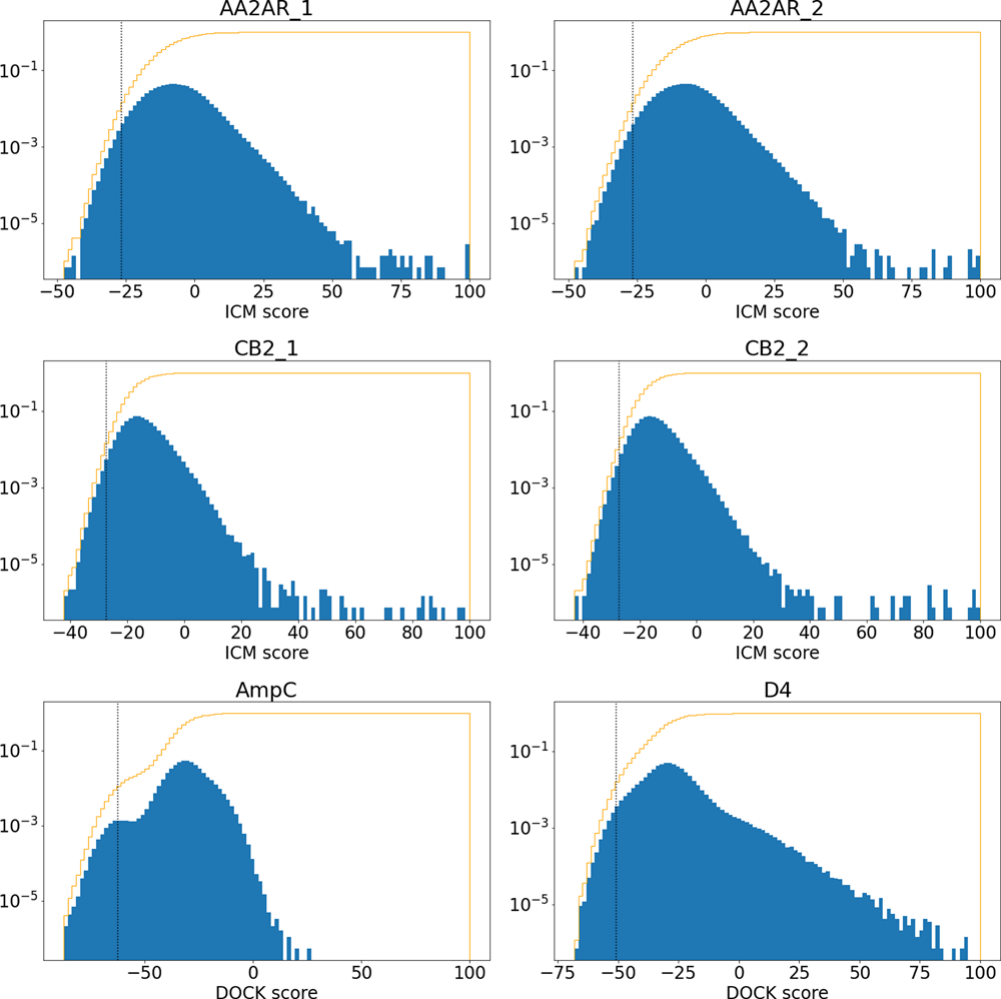
Distributions of scores for data sets used in the study. The histogram (blue) shows logscale score distribution, while the plot (orange) shows the cumulative distribution of scores. The vertical line (black) shows the cutoff for the top-1% of the scores.

For this study, we generated molecular docking scores for two targets, AA2AR and CB2 (data sets AA2AR_1/2_ and CB2_1/2_), and also used open-source data sets^9^ (AmpC and D4). For AA2AR and CB2, we used AA2AR_1_ and CB2_1_ for most of the studies in this work (and hence omit the underscore notation) and used docking scores from AA2AR_2_ and CB2_2_ as upper-bound baselines, implying that a docking itself (with a different random seed) is the best predictor for the docking result. Distribution of the scores from all six data sets is shown in Figure 1.

Using these data sets, we explored capabilities of different ML algorithms in two scenarios. First, we trained regression models on molecular fingerprints and respective docking scores and estimated the ability of regressors to predict the docking score of a molecule from its binary fingerprint. Second, we explored parameters of iterative models: after a first training-predict round, ligands with the best predicted docking score were subjected to docking again, and a new model is trained on their docking scores. We then tested these models in their ability to recover the top-1% of the ligands, ranked by their docking score.

### Molecular Docking and Fingerprint Generation

To obtain the scores for AA2AR_1/2_ and CB2_1/2_, we performed molecular docking in ICM-Pro molecular modeling software (ver. 3.9.1).^27^ We used X-ray crystal structures of CB2 (PDB ID 5ZTY, resolution 2.8 Å) and AA2AR (PDB ID 4EIY, resolution 1.8 Å). Models were prepared in accordance with the Molsoft ICM user guide.^28^ Namely, the files were loaded into the ICM-Pro package, ligands were removed, and the receptor models were converted into the ICM format using default settings, which included building of missing side chains, adding hydrogens, energy-based Gln/Asn/His conformation optimization, and removal of all water molecules. A docking box was selected within 5 Å of the cocrystallized ligands in the orthosteric pocket. As a screening library, we used randomly selected 1,000,000 drug-like (molecular weight between 200 and 500 Da, logP less than 5.0) compounds from the ZINC20 database.^25^ Using ICM-Pro, we converted compounds from SMILES to the three-dimensional SDF format, added hydrogen atoms, and assigned formal charges at pH 7.0 (according to the p*K*_a_ model implemented in ICM-Pro). Docking was performed using the ICM-Pro package (ver. 3.9-1b) without receptor flexibility and with ligand sampling thoroughness (effort) 1.0. Scoring was done with the ICM empirical scoring function.^27^ Docking score values for the best ligand pose were then obtained from the resulting SDF files.

To obtain the docking scores for AmpC and D4 targets, we used the published docking scores^9^ and randomly selected 1,000,000 molecules from the full data sets provided on Figshare.^9^ The scores, in turn, were earlier generated by the authors using the physics-based DOCK3.7 scoring function.^29^

In order to vectorize molecules for subsequent ML tasks, we generated Morgan fingerprints (size 2048, radius 2) using chemfp^30^ for all molecules from their SMILES strings. Fingerprint radius and size were chosen as a compromise between in-memory data set size and fingerprint performance in docking score prediction tasks.^19^

### Single-Iteration Retrieval of Virtual Screening Hits

First, we tested classical ML algorithms for their ability to retrieve molecules with the best docking score without any iterations (*single-iteration* regime). Namely, we labeled 1% of the top-scored ligands in data sets AmpC, D4, AA2AR_1_, and CB2_1_ as hits and the rest as nonhits. Then we tested several ML algorithms and two baseline algorithms for their ability to recover these VSHs from the ligand pool after training on a small subset of molecules with their scores. We choose a larger percentage, top-1%, compared to 0.05% in the earlier works^19,20,23^ due to the small size of the library in order to decrease the subsequent deviation of the performance metric.

In all regression problems, we used molecular fingerprints as features **X** and docking scores for target **y**, and we did not use the docking poses altogether.

As for the algorithm selection, we choose both “lightweight” (linear regression with and without regularization and support vector machine models) and “heavyweight” (random forest, decision tree, and k-neighbor models) approaches in the regression mode, predicting the docking score from the molecule’s fingerprint directly. Namely, we tested the following models (as named in the scikit-learn library; abbreviations used in figures shown in brackets): LinearRegressor (LinReg), Ridge/RidgeCV, Lasso/LassoCV, LinearSVR (LinSVR), KNeighborsRegressor (KNN), DecisionTreeRegressor (DT), and RandomForestRegressor (RF). All models were used with default parameters, as implemented in the scikit-learn Python library^31^ (ver. 0.23.2). We did not use explicit hyperparameter search, since in earlier works it showed no significant improvement in the ligand retrieval.^23^

We used 5-fold stratified cross-validation for the model performance estimation. Namely, for each train size *m*, we selected 1.25*m* ligands per each fold, labeled the top-1% as hits, and used a stratified 5-fold split to enumerate the folds. This way, models were trained on *m* ligands and tested on *m*/4 ligands, with a 1:99 imbalance of VSHs:non-VSHs in both train and test sets. Fold labels were kept the same for all models with the same train size.

For the performance measure, we used model recall: a number of true VSHs in the top-1% of hits, predicted by the model. For the AA2AR and CB2 data sets, we constructed lower- and upper-bound baselines to better understand the limits of our ML models. We used sampling from a Gaussian distribution, matching the overall score mean and standard deviation, as a lower-bound baseline (labeled “random”). A docking score from the second docking (data sets AA2AR_2_ and CB2_2_) with the same receptor was chosen as an upper-bound baseline, assuming that no ML model can provide us with a better docking score than the docking itself (labeled “Dock”). Since the docking seed was not fixed, scores were different due to the stochastic nature of the ligand sampling.^27^

### Single-Iteration Results Extrapolation

After obtaining the results for the single-iteration model performance, we explored the active learning regime. In this regime, a *base model* is trained at each step, and ligands with best scores, as this model predicts, are then docked at the next step, instead of randomly chosen ligands.

First, we estimated the effect of the batch size on the overall virtual screening performance in the active learning regime. In order to do that, we compared a few scenarios: (i) an active learning model with LinearRegression as a base model; (ii) extrapolation of a single-iteration prediction, under assumption that model’s recall remains constant; (iii) lower-bound baseline of random docking score assignment; (iv) upper-bound baseline, picking docking score from a second docking attempt (for AA2AR and CB2 data sets).

In each scenario, a base learning model was initially trained on a random batch of ligands of size *n* with their scores. This model then was used to pick the next *n* ligands from the rest of the set. Then, the next base model was trained on the scores of ligands from the previous iteration and so on.

In order to extrapolate a single-iteration model performance, we assumed that the model performance does not change with the deterioration of the ligand pool, and recall remains constant between iterations. Given the batch size *n*, total number of ligands *N*, recall *r*, and hits fraction β = 0.01, at iteration 0, the model retrieves *h*_0_ = *nβr* VSHs. At iteration *j*, the model retrieves fraction *r* of the remaining VSH: *h*_*j*_ = (*Nβ* – ∑_*i*=0_^*j*-1^*h*_*j*_)·*r*. Subsequently, the total number of hits retrieved by step *j* equals *H*_*j*_ =∑_*i*=0_^*j*-1^*h*_*j*_.

To evaluate the performance of the active learning regime, we calculated the number of retrieved VSHs. For each batch size and data set, we performed five attempts, using the same fold labels between different batch sizes. We performed calculations until 400,000 of ligands were screened, with batch sizes of 40,000, 20,000, 10,000, and 8,000 ligands, as shown in Figure 5.

### Active Learning Regime Parameters

After testing the batch size effect and the reliability of the constant recall extrapolation, we explored different scenarios of the active learning regime. Namely, we tried (i) different batch sizes (*n* = 8, 10, 20, and 40 thousand of ligands); (ii) exploiting models from earlier (*i < j*) iterations at iteration *j*; (iii) adding previously discovered ligands to the train set at step *j*, hence making the size of the train set *X*_*j*_ at step *j* equal to |*X*_*j*_| = *nj* instead of |*X*_*j*_| = *n*.

Similar to the previous section, we used here LinearRegression as a base model, given its small training and inference time, as well as its consistently good performance on all data sets. Also, for the AA2AR and CB2 data sets, we used second docking as an upper-bound baseline. Finally, we used random score assignment as a lower-bond baseline in all 4 data sets. Also, similar to the previous section, we used model recall, i.e., the amount of VSHs retrieved by the active learning model, as a performance metric.

**Figure 2:**
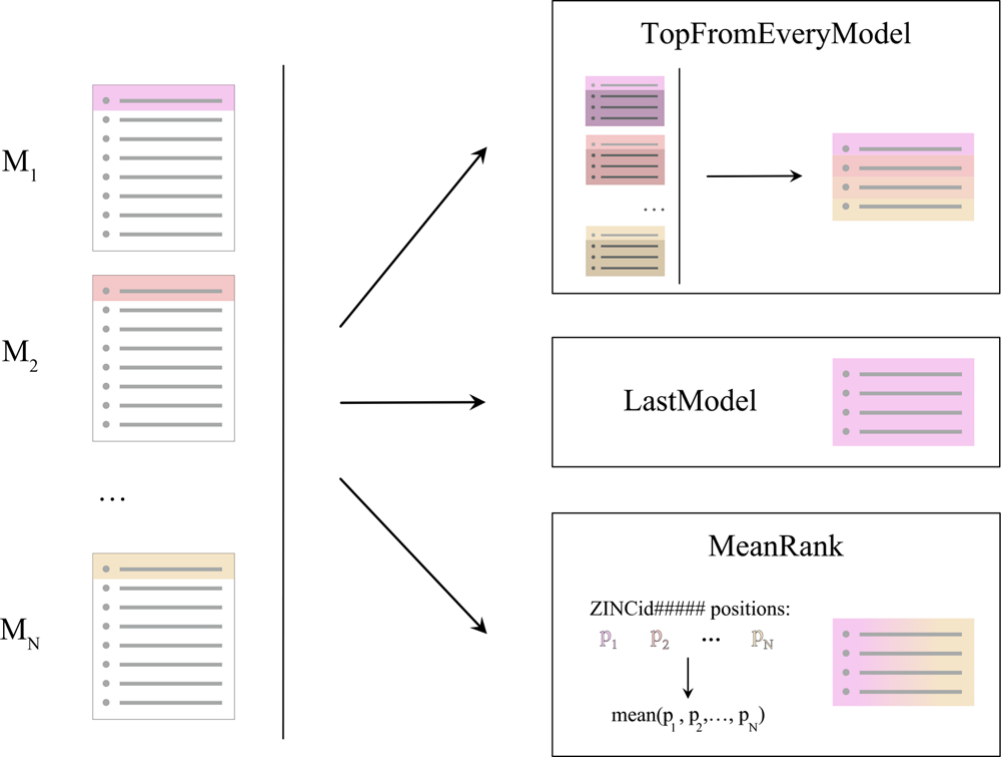
Overview of the different ensembly approaches in the active learning regime. Models *M*_1_,...,*M*_*N*_ are obtained on steps 1,...,*N* of the active learning, respectively.

We explored multiple methods to combine the results of the models trained at different iteration steps. For simplicity, we call these methods *model ensembling*, even though these models were obtained sequentially, in contrast to traditional ensembling methods in ML. Here, we used three different ensembling regimes, summarized in Figure 2. LastModel has no ensembling altogether, using only the latest model predictions. MeanRank for each ligand assigns rank *p*_*i*_ by model *M*_*i*_, trained at step *i*, and used ⟨*p_i_*⟩ as a ligand score. Finally, TopFromEveryModel used a smaller portion of each model’s top (*k* times smaller for *k* different models) and compiled individual tops into an ensemble prediction. We also explored a constant train set size at each step (“noadd” regime, as in “do not add newly available data to the train set”) versus an increasing train set size (“add” regime).

### Active Learning on an Ultralarge Library

In order to directly compare the best version of our algorithm, we performed two runs of active learning ligand retrieval using a full AmpC data set.^9^ We calculated the Morgan fingerprints (1024 bits, radius 2) using RDKit (v. 2022.3.3) for all the molecules that had docking scores present (96,214,206 ligands). We performed 100 steps of active learning ligand retrieval, with a batch size of 20,000 ligands and MeanRank ensembling regime. To simplify handling of large out-of-memory data sets and improve inference time, we reduced the number of bits (from 2048 to 1024), and also used only linear regression models trained on steps from *i* - 20 to *i* - 1 to obtain predicted scores at step *i*. For visualization of the chemical space, we used UMAP embeddings^26^ with the Jaccard metric.

### Hardware

We used a machine with 2xAMD EPYC 7502 processors at 2.35 GHz (a total of 64 cores/128 threads) and 256 Gb RAM for both docking and scikit-learn model training and inference.

## Results

### Single-Iteration Model Performance

**Figure 3:**
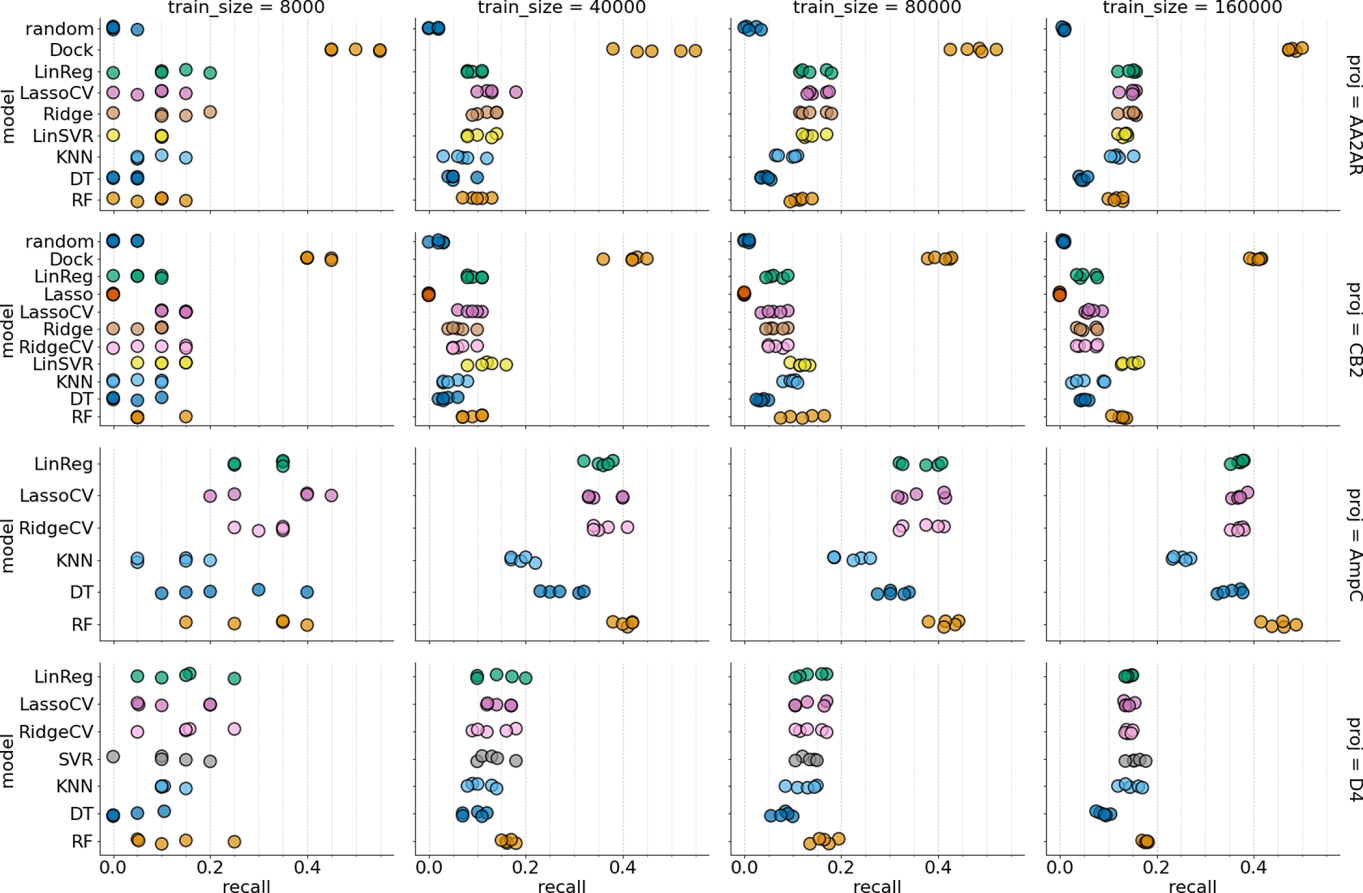
Model performance for multiple regression models and their baselines on four data sets present in the study. Rows represent different data sets, and columns show different training sizes. Values for five independent folds are shown.

Figure 3 shows that the tested models have performed differently on different data sets assessed in this study. Namely, for the data sets where the scores were obtained with DOCK (D4 and AmpC), recall scores tend to be higher than for those obtained with ICM.

Also, the increase in the training data set size does not lead to significant improvements in the model’s performance, in line with previously observed results.^23^ Despite that, “heavyweight” models such as RF seem to benefit from that more, compared to more “lightweight” linear models, which seem to saturate their performance at a train size of around 80,000. Interestingly, adding regularization to linear models does not increase their performance, as seen by LassoCV and RidgeCV performance.

**Figure 4:**
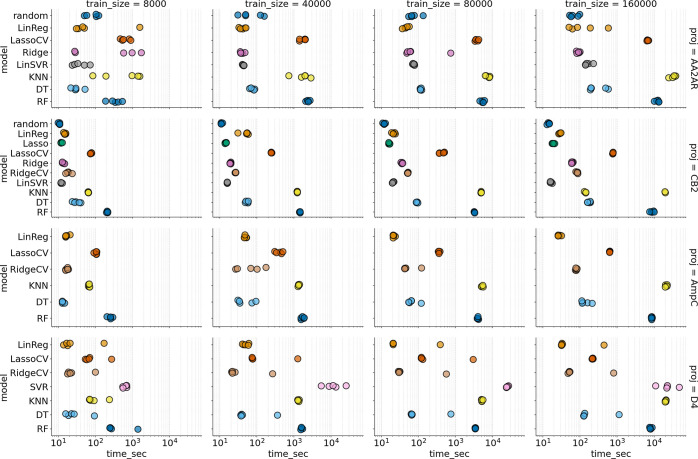
Execution time (log scale) of the train-predict loop of the algorithms evaluated in the single-iteration regime. Rows represent different data sets, and columns show different training sizes. Values for five independent folds are shown.

Notably, the execution time (Figure 4) for the linear regression remains within few minutes, whereas more “heavyweight” algorithms take up to 100 times more for the train-predict loop even on a million-sized library, while previous works reported up to a day of train-predict time using modern GPUs.^15,21^

### Extrapolation of Single-Iteration Results

**Figure 5:**
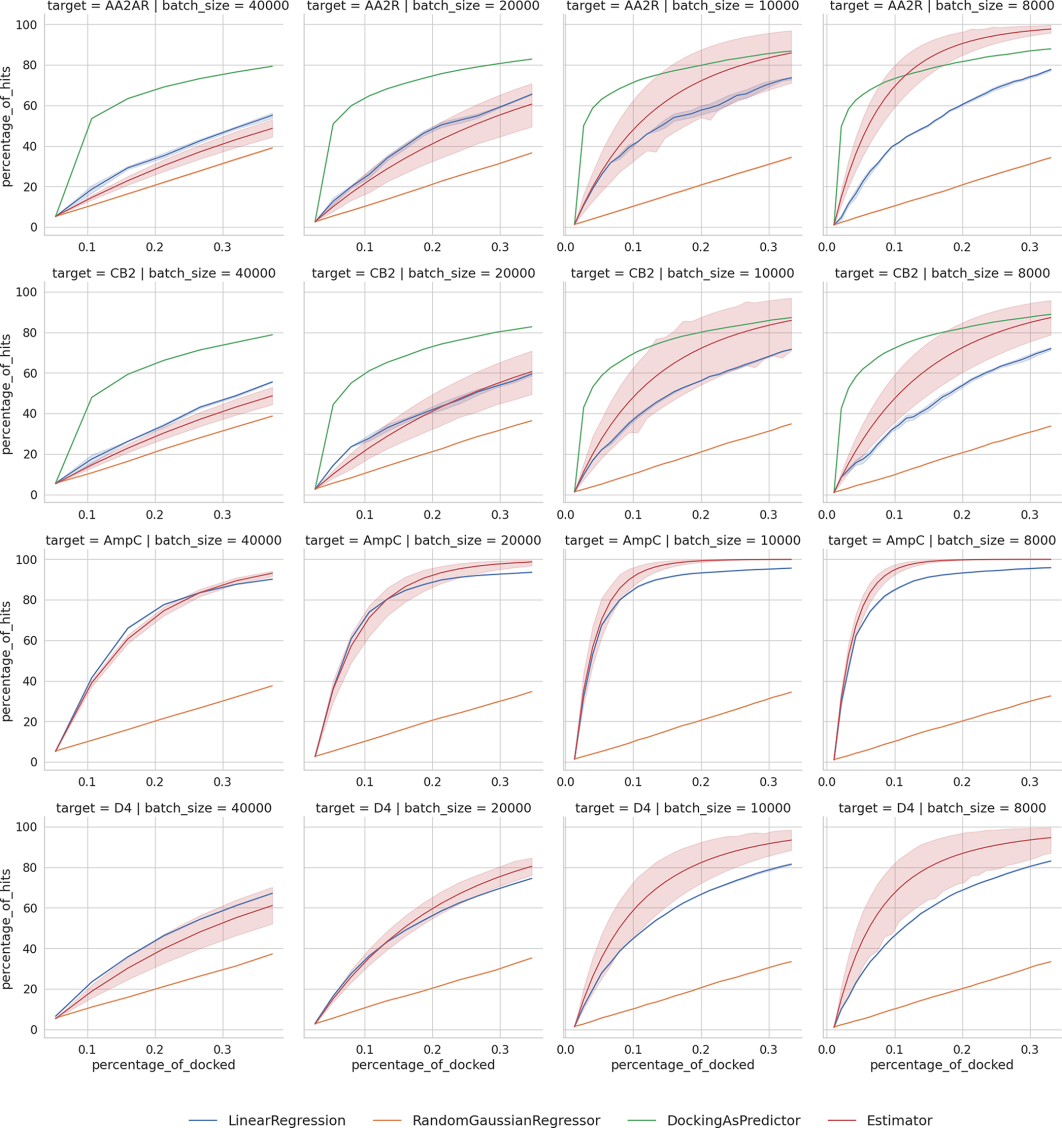
Comparison of the single-iteration extrapolation with the simplest active learning model. Plots show cumulative percentage of hits vs percentage of the library docked, for different regimes: actual active learning model (blue), random docking score assignment baseline (orange), using independent docking run to obtain upper-bound baseline (green), and extrapolation from a single-iteration performance of a LinReg base model (red).

Following the robust performance of LinReg in the single-iteration regime, we compared the active learning regime with extrapolation from single-iteration performance, as summarized in Figure 5. It is clear that for the large batch size (40,000 or 20,000), extrapolation can reliably predict an outcome of the active learning. However, with the decrease of the batch size, extrapolation seems to be overestimating the performance. Despite that, the superior performance of active learning with smaller batch size is still obvious.

Since at each step only around 10–15% of the retrieved molecules belong to the top-1% (Figure 3), the rest of the test set is comprised of molecules with lower scores. Hence, we believe that smaller batch sizes help to prevent overfitting and enrich the retrieved molecule pool with more diverse molecules at each step, thereby providing an efficient exploration of the chemical space, whereas larger batch sizes retrieve similar molecules at each step, and with a smaller number of steps, less chemical diversity is achieved.

**Figure 6:**
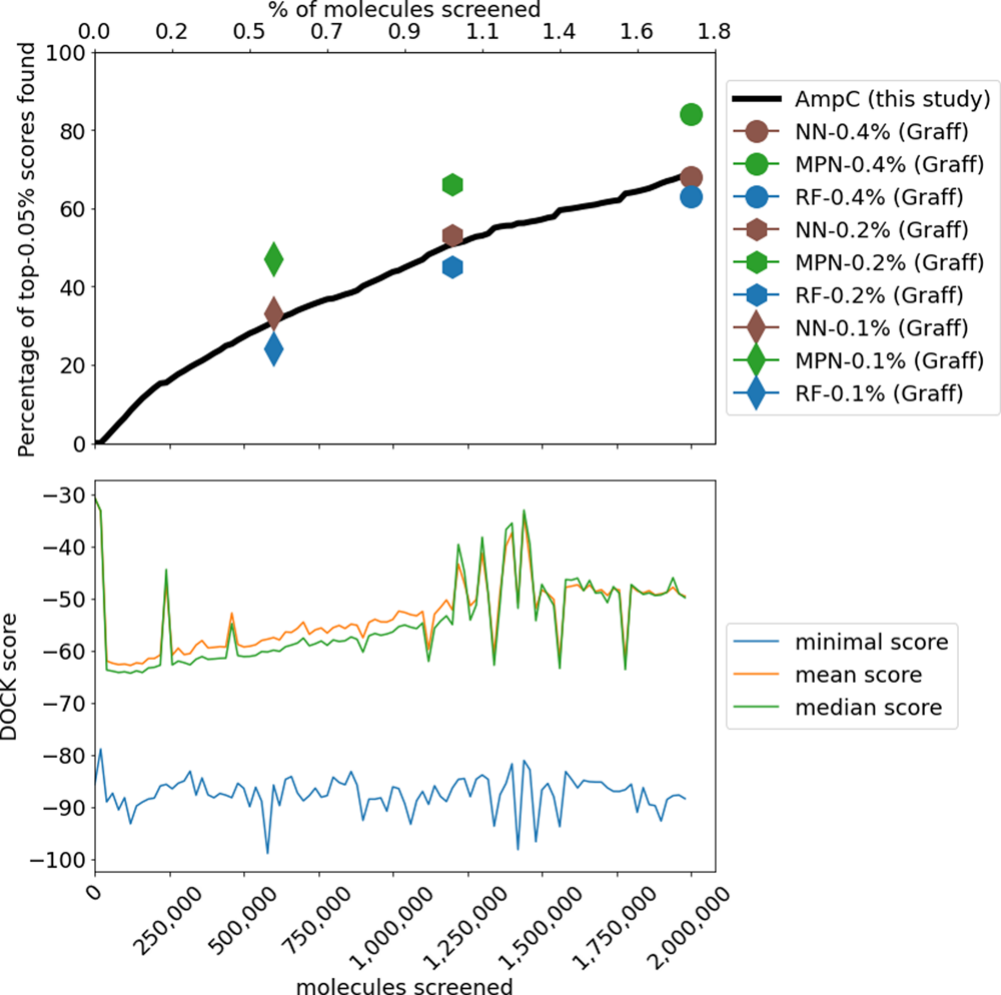
Comparison of the best active learning model, designed in this study, with results from Graff et al.^19^ The solid line shows the percentage of the top-0.05% of ligands found after screening a certain amount of ligands. Lines on the lower subplot represent minimal, mean, and median scores of each iteration batch.

It is worth noting that our results of the simple active learning model go in line with the previously reported results. Namely, in Graff et al.^19^ (as shown here in Figure 5), after exploring six hundred thousand molecules, models with a smaller batch size (0.1%) consistently find more molecules than those with larger batch sizes (0.2% and 0.4%). We believe that the absolute number of docked ligands, and not the number of active learning steps, is a more suitable scale for this case (see also Figure 6), since the docking itself, especially in our case of lightweight models, is the most time-consuming step.

Interestingly, when a second docking run is used as a docking score predictor, it demonstrates superior performance in the first few iterations, but then its predictions yield fewer real hits per step than the random search, as shown in Figure 5 (data sets AA2AR and CB2). This is likely happening since the reliably predicted VSHs are quickly exhausted in the first few steps, and the second docking is effectively useless for ligands with lower scores. Potentially, comparing the success of the active learning batch prediction with the random batch prediction might serve as a stopping criterion for the real accelerated docking screening campaign.

### Optimal Parameters of the Active Learning Regime

The meta-parameters of the active regime focused on sharing information about the docking results between different batches as well as increasing the training size between batches. Here, we discuss the *early* recall (percentage of VSHs obtained after docking approximately 10% of the library) and *late* recall (after docking 30% of the library).

As summarized in Table 1, models perform drastically differently with different data sets: while for the AmpC data set, around 90% of the VSHs are found already after screening the first 10%, and for the CB2 data set, even the best late recall is around 80%, also requiring three times as many ligands docked. Besides, the relative model performances between different data sets are clearer.

**Table 1:** Recall Score of the Early Stage (after 10% Library Screened) and Late Stage (after 30% Library Screened)^a^

			Acquired hits, %							
			AA2AR		CB2		AmpC		D4	
Compounds docked, %	Batch size, k	Ensembling	“add”	“noadd”	“add”	“noadd”	“add”	“noadd”	“add”	“noadd”
10	40	“LastModel”	18 ± 4	19 ± 4	17 ± 5	17 ± 5	41.2 ± 0.2	41.6 ± 0.1	23.3 ± 0.5	23.5 ± 0.3
		“MeanRank”	18 ± 4	18 ± 4	17 ± 5	17 ± 5	41.3 ± 0.3	41.2 ± 0.2	23.3 ± 0.5	23.4 ± 0.2
		“TopFromEveryModel”	18 ± 3	18 ± 4	17 ± 5	17 ± 5	41.1 ± 0.2	41.2 ± 0.2	23 ± 1	23.3 ± 0.5
		Second docking	53.8 ± 0.4	53.8 ± 0.2	47.57 ± 0.01	47.7 ± 0.3	NA	NA	NA	NA
	20	“LastModel”	20 ± 8	26 ± 4	31.4 ± 0.3	28 ± 3	74.4 ± 0.4	74 ± 1	37.9 ± 0.1	36.35 ± 0.03
		“MeanRank”	30 ± 7	28 ± 5	29.8 ± 0.5	30.5 ± 0.3	70 ± 1	72 ± 1	36.6 ± 0.2	37.8 ± 0.5
		“TopFromEveryModel”	26 ± 8	25 ± 7	27 ± 2	28 ± 2	69.6 ± 0.4	70.6 ± 0.4	36.0 ± 0.3	35.6 ± 0.3
		Second docking	64.8 ± 0.2	64.9 ± 0.2	61.1 ± 0.2	60.9 ± 0.3	NA	NA	NA	NA
	10	“LastModel”	50 ± 2	42.1 ± 0.4	43 ± 3	39 ± 2	90.2 ± 0.1	87 ± 1	55.4 ± 0.3	46.7 ± 0.4
		“MeanRank”	45 ± 4	48 ± 4	41 ± 1	44 ± 1	85 ± 1	88.6 ± 0.3	51.6 ± 0.4	54.0 ± 0.5
		“TopFromEveryModel”	47 ± 2	43 ± 1	39 ± 6	36 ± 1	86 ± 1	85.3 ± 0.3	50.7 ± 0.3	47.0 ± 0.3
		Second docking	72.25 ± 0.03	72.3 ± 0.1	70.4 ± 0.1	70.61 ± 0.03	NA	NA	NA	NA
	8	“LastModel”	45 ± 7	42 ± 1	40 ± 20	34 ± 3	92.4 ± 0.1	86 ± 1	61.8 ± 0.1	48.3 ± 0.3
		“MeanRank”	40 ± 10	51 ± 3	39 ± 16	48 ± 3	88 ± 1	91.3 ± 0.4	56.7 ± 0.5	60 ± 1
		“TopFromEveryModel”	40 ± 10	42 ± 3	40 ± 13	38 ± 1	89.2 ± 0.2	87.8 ± 0.3	56 ± 1	49.5 ± 0.4
		Second docking	74.07 ± 0.02	74.1 ± 0.1	73.1 ± 0.1	73.2 ± 0.1	NA	NA	NA	NA
30	40	“LastModel”	58 ± 2	55 ± 3	51 ± 4	56 ± 1	89.1 ± 0.3	90.2 ± 0.4	67.7 ± 0.3	67.2 ± 0.2
		“MeanRank”	58 ± 4	56 ± 4	51 ± 5	53 ± 3	86.8 ± 0.5	87.8 ± 0.5	67 ± 1	68.0 ± 0.3
		“TopFromEveryModel”	54 ± 6	57 ± 2	51 ± 7	53 ± 5	87.3 ± 0.2	88.4 ± 0.3	67 ± 1	67.1 ± 0.1
		Second docking	79.7 ± 0.4	79.5 ± 0.2	78.5 ± 0.5	78.0 ± 1.0	NA	NA	NA	NA
	20	“LastModel”	50 ± 15	66 ± 1	64 ± 2	60 ± 2	94.19 ± 0.02	93.6 ± 0.0	76.1 ± 0.5	74.5 ± 0.4
		“MeanRank”	62 ± 14	66 ± 3	63 ± 3	65 ± 3	92.3 ± 0.3	93.2 ± 0.4	74.8 ± 0.2	75.6 ± 0.4
		“TopFromEveryModel”	63 ± 5	65 ± 3	63 ± 2	63 ± 2	93.1 ± 0.3	94.3 ± 0.2	74.6 ± 0.1	74.4 ± 0.4
		Second docking	82.9 ± 0.2	82.7 ± 0.2	83.0 ± 0.1	82.9 ± 0.2	NA	NA	NA	NA
	10	“LastModel”	79 ± 2	74 ± 2	73 ± 2	71.6 ± 0.4	97.0 ± 0.1	95.6 ± 0.1	85.6 ± 0.3	81 ± 1
		“MeanRank”	70 ± 10	82 ± 1	73 ± 4	76.8 ± 0.2	96.2 ± 0.4	96.2 ± 0.2	84.3 ± 0.1	84.8 ± 0.2
		“TopFromEveryModel”	78 ± 7	78 ± 1	68 ± 10	72 ± 1	96.8 ± 0.1	97.0 ± 0.1	84.1 ± 0.1	81.8 ± 0.2
		Second docking	86.68 ± 0.01	86.80 ± 0.02	87.3 ± 0.1	87.5 ± 0.2	NA	NA	NA	NA
	8	“LastModel”	76 ± 10	78 ± 2	70 ± 20	72 ± 2	97.5 ± 0.1	96 ± 1	88.1 ± 0.4	83.1 ± 0.3
		“MeanRank”	75 ± 6	85 ± 1	70 ± 20	80.3 ± 0.4	96.9 ± 0.1	96.8 ± 0.1	86.7 ± 0.1	87.6 ± 0.3
		“TopFromEveryModel”	77 ± 5	80 ± 2	69 ± 15	75 ± 1	97.3 ± 0.1	97.5 ± 0.1	86.9 ± 0.2	84.0 ± 0.1
		Second docking	88.1 ± 0.1	88.0 ± 0.1	88.8 ± 0.3	88.89 ± 0.01	NA	NA	NA	NA

a Errors represent standard deviation within five independent folds. “add” and “noadd” labels represent gradual increase of the train set versus constant train set size.

For all meta-parameters, the decrease of the training size is also beneficial here, in agreement with the extrapolation of single-iteration results. For different data sets, the smallest vs largest batch size (40,000 vs 8,000) results in 2–3 times difference in the early recall, although difference in the late recall is less significant.

Adding simple information exchange between different iterations steps via MeanRank ensembling mechanism or via simply increasing train size (LastModel with “add” parameter) confidently boosts performance: both early and late recall is 10–15% points higher, compared to the LastModel-noadd regime. Interestingly, using TopFromEveryModel ensembling does not boost the performance compared to the LastModel regime. We suggest that this happens due to the inaccuracy of each independent model’s top 1%, as previously seen in Figure 3.

However, differences in the model performance with different ensembling mechanisms are less obvious. If we focus on the “noadd” regime that keeps the training size constant between the batches, we can see that at batch size 8,000, ensembling regime MeanRank has better performance compared to the two other methods. In this regime, each base model learns a piece of valuable information about its chemical subspace, and low-ranked molecules from a single base model can still end up in the final list of hits for the next iteration.

Interestingly, a gradual increase of the train size does not boost the overall performance of the active learning regime with any kind of ensembling. It agrees well with the single-iteration performance results and the choice of simple LinearRegression models, which seem to saturate in their performance, as observed before.

Also, even though the LastModel regime shows a worse performance compared to others, it is still considerably higher than the performance of the random choice screening. For example, for the least performative CB2 data set, it still finds around 34% of the VSHs after screening only 10% of ligands, around half of the VSHs for D4 and AA2AR data sets, and 86% VSHs for the AmpC data set (Table 1).

### Active Learning on an Ultralarge Library

Figure 6 demonstrates results of the ultralarge data set active learning using the full AmpC data set. Despite the simplicity of the active learning model (MeanRank ensembling of 20 independently trained linear regression models at the last 20 steps), its performance is on par with the deep learning architectures, such as multilayer perceptron or the message-passing neural network (“NN” and “MPN”, respectively). Notably, a simple ensemble of linear regressions consistently outperforms a random forest architecture despite its training set size (labels 0.1%, 0.2%, and 0.4%). Given that in our benchmarks, RandomForest models were around 100 times slower to train, this shows that linear regression models with a simple ensembling mechanism may outperform more complex machine learning models in both training time and performance.

**Figure 7:**
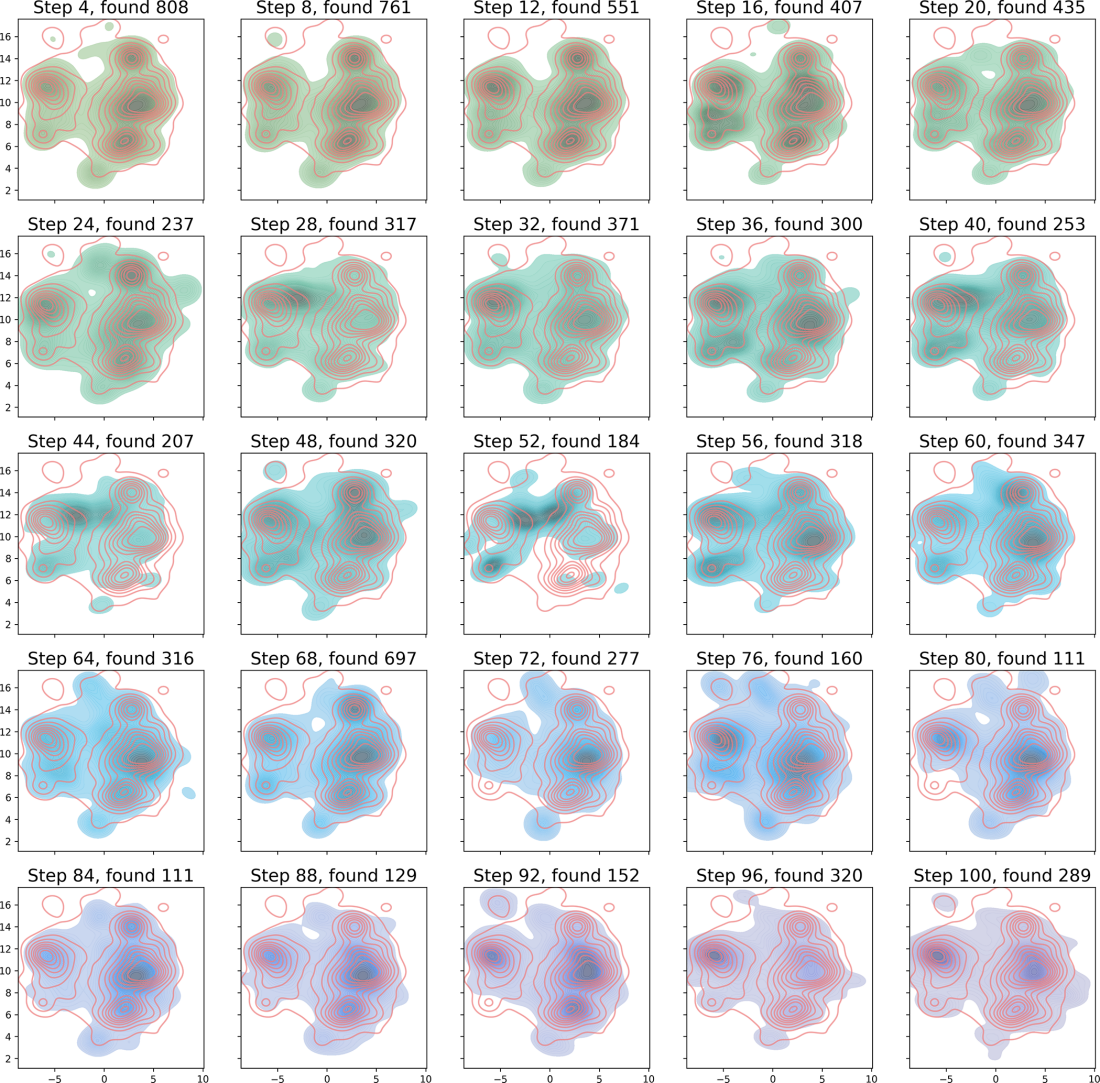
Representation of the chemical space at 25 different steps of active learning. Both the *x* and *y* axes correspond to the UMAP embeddings. Red contours show the density plot for the top-0.05% of the library, while colored plots show the density for the actually retrieved ligands. The number of ligands found at each iteration is shown at the top of each subplot.

Interestingly, the simplicity of the linear regression as a base model provides greater chemical diversity in the retrieved ligands. Figure 7 shows the UMAP representation of the chemical space, represented by Morgan fingerprints, at different active learning steps. It is clear that different iterations focus on different parts of the chemical space (e.g., iteration 40 and iteration 60) while remaining close to the top-0.05% of the ligands. Moreover, clear differences in the retrieved chemical space embeddings with the top-0.05% ones result in a low number of retrieved ligands (e.g., iteration 52). The diversity of the ligands can also indirectly be observed by the fluctuations in the ligand docking score (Figure 6), showing that some batches are clearly different from the top of the ligand list.

## Discussion

In this work, we propose a domain-specific active learning model for acceleration of the docking-based ultralarge library screening. Compared to previous works in the field,^15,19−21,23,24^ we focus on different aspects of this approach, such as ML model complexity, and influence of information exchange between models trained on different docking batches.

Given exponentially growing chemical databases,^32,33^ we focus here on performance of the simplest models such as linear regression, that are computationally efficient for both training and inference on extremely large data sets. Robust performance of the linear methods shown here allows one to use vector databases^34−36^ for the precomputed fingerprints and extract and compute molecule 3D structures and subsequently dock them only when needed, thus drastically reducing the computational requirements for the actual docking.

As shown by comparison with the second docking for CB2 and AA2AR data sets, ligand docking (especially with a low sampling depth) is relatively inaccurate and can retrieve only half of the ligands from the data set in the active learning regime after docking of the first 10%. We believe that this intrinsic inaccuracy of this method is what gives the simple linear models such robustness in this case. Due to the limited sampling depth, the docking scores are not accurate enough, so that any complex model will not be able to predict their true value. Hence, model complexity can be reduced drastically without losing its performance.

Also, we note the importance of a random train-test split of the molecules, even though for the chemical data sets it is often important to use a similarity-aware data split. For small-molecule docking, chemically similar molecules often have similar docking scores and even require clustering after the screening to select hits for experimental validation. Hence, a machine learning model’s task is to find similar molecules to the already docked ones with high docking score. However, simple chemical similarity is not enough for this task, as illustrated by poor performance of the nearest neighbors models (Figure 3). However, a linear regression can learn the most important fingerprint bits and reliably extract respective molecules for the subsequent docking.

Even though for the linear regression the model inference is fast, the actual active learning regime that incorporates information from earlier iterations might substantially increase the inference time for the later stages of the screening, when the number of models for ensembling is large. However, in this work, we show that the simple MeanRank regime that averages molecule ranks for each model shows superior performance. Fortunately, for this type of ensembling, a base model’s results can be cached for later, without the need to rerun the model inference at each stage.

Moreover, here we show that state-of-the-art performance can be achieved even without the need for GPU computational resources, which were employed by all but one previous works either in the message-passing neural networks,^19^ as DeepChem models,^23^ or as multilayer perceptron.^21^ This can significantly increase the availability of the method. Compared to the Martin et al.,^20^ who also focus on reducing the accelerated virtual screening costs, we show that incorporation of simple ensembling mechanisms into the active learning model can substantially increase its performance on both early and late stages of the screening.

We compare our best model using data sets known in the field and show that its performance is similar to the more computationally requiring deep learning and classical machine learning models–namely, it retrieves about 70% of the top-0.05% of the AmpC library after screening only 2% of the molecules, while the best deep learning model (message-passing neural network) retrieves 84%, and other models (random forest and multilayer perceptron) retrieve less than 70% of the molecules. We show that our weak models explore chemical space without significant signs of overfitting or preference to its small subspace, which logically results in retrieval of most of the top-0.05% of the ligands in the ultra large-scale benchmark.

Interestingly, our work outlines the importance of the batch size effect in chemical space exploration. Namely, a smaller batch size tends to outperform a larger one in the active learning regime across all ensembling regimes. Moreover, if we check the same effect in Graff et al.,^19^ all three “base” algorithms trained on a smaller batch size retrieve more virtual hits after an equal amount of ligands screened, although after more active learning iterations. We speculate that a small enough batch size in the active learning regime is crucial for a better algorithm performance, although its previse value is obviously dependent on the nature of the docking algorithm and a screened library. For instance, for a chemically diverse library, one would expect more complex models, such as MPN, to perform better, being able to learn properties that are inaccessible with simple binary fingerprints. On the contrary, in chemical libraries with high enough similarity between compounds, such as Enamine, a simple linear regression would be able to find core properties of highly scored clusters that usually appear in a VLS campaign,^5^ neglecting the need for a more computationally expensive model.

Finally, choosing linear regression over more heavyweight models as the active learning “base” algorithm depends on the proportion between three consecutive steps: time spent on docking of a single batch, subsequent model training, and its inference on the unexplored part of the library. Prior to our work, model training and inference times were the bottlenecks for the single iteration; hence, reducing them with little effect on performance turned out to be productive in real-world scenarios. However, choosing a much more heavyweight docking algorithm can shift the proportion toward docking itself, making the benefit of having fast model training and inference negligible compared to the benefit of slightly better model performance.

## Conclusion

In this work, we demonstrate that linear regression based active learning is well suited for the accelerated screening of ultralarge virtual libraries via structure-based docking. Using a few diverse data sets as an example, we show that linear models, such as linear regression, show performance comparable with much more computationally requiring models, such as random forest or decision tree. Our benchmarks demonstrate that models with a small batch size of 10,000 molecules perform better at the active learning regime, which decreases the potential model training requirements and time. We show a substantial decrease in computational time, retrieving, for various data sets, 48–91% of the top-1% of the ligands after docking 10% of the library and 85–98% after docking 30% of the library. We hypothesize that such a robust performance of linear models is coupled with intrinsic inaccuracy of the low sampling depth of small molecule docking. We prove the viability of our model in an ultralarge-scale virtual screening benchmark, showing performance comparable to deep learning models that require a single day for training and inference on modern GPUs. We propose that the choice of the “base” model for an active learning large-scale virtual ligand screening campaign depends on the nature of the screened chemical library (less similarity–simpler models) and available computational resources. Finally, we envision the wider application of active learning agents based on linear regression that will greatly democratize access to this approach for the academic community.

## Data Availability

Docking scores, obtained with ICM-Pro and used in this study, are available in the repository https://github.com/marinegor/Linear-accelerated-docking. We used Molsoft ICM 3.9-1b for docking and chemfp 1.6.1 for fingerprint preparation in a single-iteration regime and rdkit v. 2022.3.3 for fingerprint generation in an active learning regime. We used sklearn 0.23.2 for model training and inference. The active learning benchmark code is available at https://github.com/marinegor/Linear-accelerated-docking.
